# Activation of ERAD Pathway by Human Hepatitis B Virus Modulates Viral and Subviral Particle Production

**DOI:** 10.1371/journal.pone.0034169

**Published:** 2012-03-26

**Authors:** Catalin Lazar, Alina Macovei, Stefana Petrescu, Norica Branza-Nichita

**Affiliations:** Institute of Biochemistry of the Romanian Academy, Department of Viral Glycoproteins, Bucharest, Romania; Yonsei University, Republic of Korea

## Abstract

Hepatitis B virus (HBV) belongs to the *Hepadnaviridae* family of enveloped DNA viruses. It was previously shown that HBV can induce endoplasmic reticulum (ER) stress and activate the IRE1-XBP1 pathway of the unfolded protein response (UPR), through the expression of the viral regulatory protein X (HBx). However, it remained obscure whether or not this activation had any functional consequences on the target genes of the UPR pathway. Of these targets, the ER degradation-enhancing, mannosidase-like proteins (EDEMs) are thought to play an important role in relieving the ER stress during UPR, by recognizing terminally misfolded glycoproteins and delivering them to the ER-associated degradation (ERAD). In this study, we investigated the role of EDEMs in the HBV life-cycle. We found that synthesis of EDEMs (EDEM1 and its homologues, EDEM2 and EDEM3) is significantly up-regulated in cells with persistent or transient HBV replication. Co-expression of the wild-type HBV envelope proteins with EDEM1 resulted in their massive degradation, a process reversed by EDEM1 silencing. Surprisingly, the autophagy/lysosomes, rather than the proteasome were involved in disposal of the HBV envelope proteins. Importantly, inhibition of the endogenous EDEM1 expression in HBV replicating cells significantly increased secretion of both, enveloped virus and subviral particles. This is the first report showing that HBV activates the ERAD pathway, which, in turn, reduces the amount of envelope proteins, possibly as a mechanism to control the level of virus particles in infected cells and facilitate the establishment of chronic infections.

## Introduction

Hepatitis B virus (HBV) is a noncytopathic, hepatotropic virus which belongs to the *Hepadnaviridae* family. HBV infection is a serious health problem, resulting in acute and chronic hepatitis, cirrhosis and often hepatocellular carcinoma and death [1], [2]. Despite the existence of an efficient vaccine, more than 400 million people are known to carry the virus worldwide.

The viral DNA genome is packaged inside the nucleocapsid, surrounded by a lipid bilayer derived from the host cell, which contains three transmembrane proteins translated from alternative start codons of the same open reading frame (ORF). These surface proteins are designated as large (L), middle (M) and small (S) and share a 226 amino acid- long S domain, at the C-terminal region [3]. In addition to the S domain, the M protein contains a 55 amino acids pre-S2 region, also present in L. The L protein is further extended by an N-terminal pre-S1 domain comprising 109 amino acids. The envelope proteins are translocated into the endoplasmic reticulum (ER) where N-glycosylation, folding and oligomerization occur [4]. This compartment is also responsible for the quality control of the newly synthesized proteins, which ensures the disposal of polypeptides failing to fold through the calnexin/calreticulin cycle [5]. Terminally misfolded proteins are retro-translocation into the cytosol, followed by polyubiquitylation and proteasomal degradation [6]. This tightly regulated ER-associated degradation (ERAD) pathway is initiated by the oligomerization and autophosphorylation of the ER stress-sensor IRE1, which, once activated, removes an intron from the X-box binding protein 1 (XBP1) mRNA [7].

The spliced mRNA is translated into an efficient transcription factor which triggers the expression of proteins and enzymes of the ER degradation-enhancing, mannosidase-like proteins (EDEM) family [8].

EDEMs are believed to function as lectins that recognize terminally misfolded glycoproteins and deliver them to the ERAD pathway, thus relieving the ER stress resulted from their accumulation [9], [10], [11].

It was previously suggested that HBV may induce ER stress and activate the IRE1-XBP1 pathway of the unfolded protein response (UPR), through the expression of the viral regulatory protein X (HBx) [12]. However, it was not clear, whether the target genes of this pathway were correspondingly activated. A more recent study has shown that over-expression of the S protein activates the cellular autophagy, a process which is beneficial for HBV replication, but also triggers the UPR, suggesting a potential implication of the latter pathway in the viral life cycle [13]. However, the consequences of the UPR activation on either the infected-host cell or the virus remained largely obscure.

In this study we investigated the role of the ERAD pathway in modulating the HBV life cycle and production of viral and subviral particles (SVPs). We found that synthesis of the EDEM family of proteins (EDEM1 and its homologues, EDEM2 and EDEM3) is significantly up-regulated in cells with persistent or transient HBV replication. Interestingly, co-expression of the wild-type S, M and L proteins with EDEM1 resulted in massive degradation of the envelope proteins, which was reversed by EDEM1 silencing. Degradation occurs before protein oligomerization in native complexes, by autophagy/lysosome. Inhibition of endogenous EDEM1 expression in HBV replicating cells increased secretion of enveloped virus as well as SVPs.

This is the first report showing that HBV activates the ERAD pathway, which, in turn, reduces the amount of envelope proteins, possibly as a mechanism of controlling the level of virus particles in infected cells and facilitate the establishment of chronic infections.

## Results

### HBV activates expression of EDEM proteins

**Figure 1:**
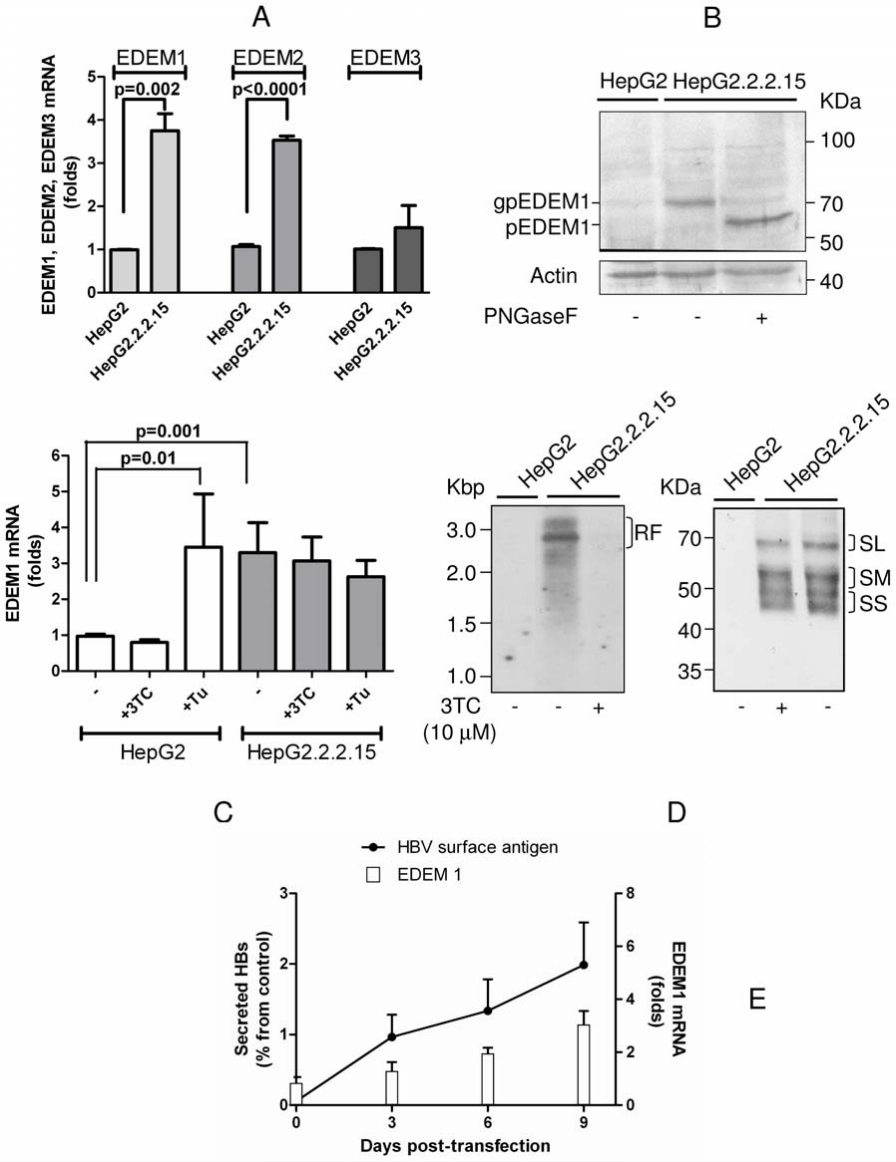
EDEMs are up-regulated in HBV-replicating cells. (A) The levels of EDEM1-3 mRNA were quantified by RT-real-time PCR, in HepG2 and HepG2.2.2.15 cells. (B) EDEM1 expression was investigated in HepG2 and HepG2.2.2.15 cells, by Western blotting, following PNGase F digestion. Glycosylated (gp) and non-glycosylated (p) EDEM1 are shown. Actin expression was used as total protein gel-loading control. (C) The level of EDEM1 mRNA was quantified by RT-real-time PCR, in HepG2 and HepG2.2.2.15 cells, in the absence or presence of lamivudine (3TC) or tunicamycin (Tu). (D) Quantification of HBV replication and envelope protein biosynthesis by Southern blotting (left panel) and Western blotting under non-reducing conditions (right panel). The replication forms (RF) and the envelope protein oligomers are shown. (E) The amount of SVPs and EDEM1 mRNA were quantified pTriexHBV1.1-transfected Huh7 cells, by ELISA and RT-real-time PCR, respectively. Data represent the mean and standard deviation (SD) of duplicates from three independent experiments. The cut-off values in ELISA varied between 0.082–0.095. Statistical analysis showing “p” values was performed using the Student's unpaired t-test (A, C).

To investigate the relationship between HBV replication and EDEM proteins expression, the level of EDEM1-3 transcripts was quantified in HepG2.2.2.15 cells, which support active HBV replication, assembly and secretion of infectious virions, and the parental HepG2 cell line. Interestingly, an important increase in the amount of EDEM1 and EDEM2 mRNA (approximately 4 fold) was observed in HepG2.2.2.15 compared to HepG2 cells, while EDEM3 mRNA increased only moderately (by 2 fold) (Fig. 1A). To further determine whether the increased level of mRNA was accompanied by an accumulation of the corresponding proteins, EDEM1-3 biosynthesis was analysed by Western blotting of both cell lines lysates, following digestion with PNGase F. Expression of endogenous EDEM2 and EDEM3 was not detectable on Western blots in either cell line, although both, commercial as well as home-made Abs were used (data not shown). In contrast, endogenous EDEM1 was readily detectable in HepG2.2.2.15, while only a faint band was observed in HepG2 cells, confirming the results obtained at mRNA level (Fig. 1B). EDEM1 identity was further confirmed by PNGase F treatment which decreased the apparent molecular size of the 78 kDa glycosylated protein (gp) to that of the fully deglycosylated, 69 kDa polypeptide (p), as expected [11].

Since endogenous EDEM1 was expressed as sufficiently high levels to be detectable in HepG2.2.2.15 cells, the next experiments were focused on the role of this protein, as a representative of the EDEM family, in the HBV life-cycle.

Over-expression of the envelope S protein was previously shown to induce UPR [13]. To determine whether accumulation of nucleocapsids contributes to the significant up-regulation of the EDEM proteins expression in HepG2.2.2.15 cells, the amount of EDEM1 mRNA was quantified in the presence of the HBV replication inhibitor, lamivudine (3TC). Tunicamycin (Tu), an N-glycosylation inhibitor known to induce UPR [14] was also included as positive control for the ER stress. As shown in Fig. 1C, 3TC treatment had no impact on the level of EDEM1 transcripts. The efficiency of the viral replication inhibition was demonstrated by Southern blotting, which revealed a dramatic decrease of the amount of HBV nucleocapsids in 3TC-treated cells, by more than 95% (Fig. 1D, left panel). The drug had, however, no significant effect on the HBV envelope proteins, as shown by Western blotting of the same cell lysates (Fig. 1D, right panel). This is expected, since viral protein synthesis occurs also independently of replication in HepG2.2.2.15 cells, from the transcripts generated by the nuclear copies of the HBV genome. Together, the results show that accumulation of viral nucleocapsids is not responsible for the up-regulation of EDEM1 transcripts.

Interestingly, Tu increased the EDEM1 mRNA amount in HepG2 cells to a level similar to that found in HepG2.2.2.15 cells, in the absence of any treatment, while having no consequences on the steady-state level of EDEM1 mRNA in HepG2.2.2.15 cells. This suggests that unlike HepG2, the HepG2.2.2.15 cells are already exposed to a significant amount of chronic ER stress, due to the high rate of HBV replication, to which they have adapted by eliciting a sufficient level of UPR [12].

To determine the relationship between HBV and EDEM1 expression in cells hosting a progressive HBV replication, the EDEM1 transcripts were quantified in Huh7 cells transfected with pTriex HBV 1.1. The plasmid contains 1.1 units of the whole HBV genome and is able to support viral replication, assembly and secretion of fully infectious virions [15]. As shown in Fig. 1E, the amount of EDEM1 mRNA correlated well with that of HBV protein synthesis, with a maximum level at day 9 post-transfection (up to 3 fold increase).

### EDEM1 enhances degradation of HBV envelope proteins

**Figure 2:**
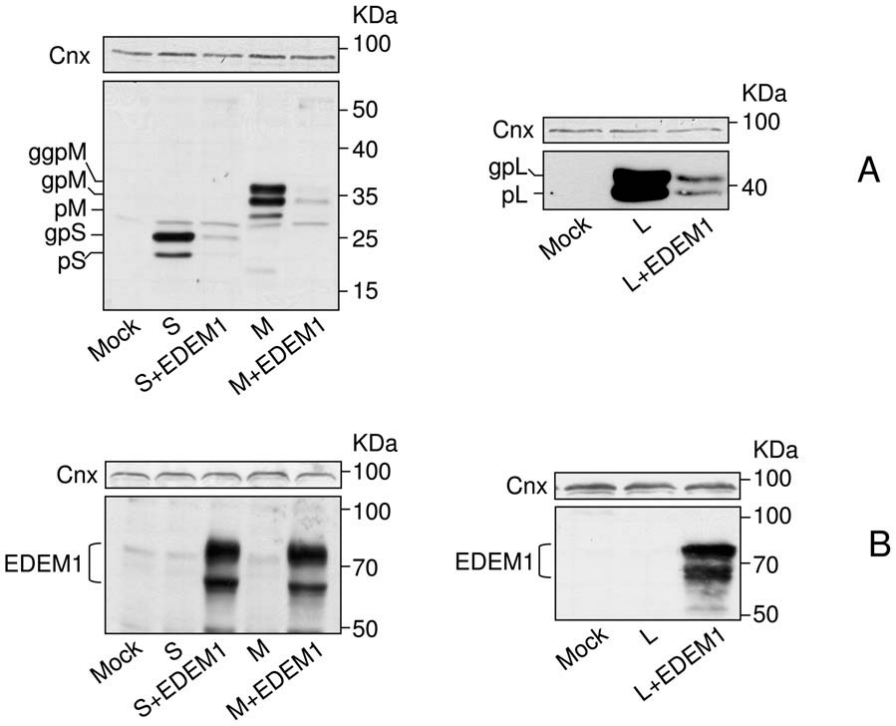
EDEM1 over-expression results in degradation of the HBV envelope proteins. HEK293T were transfected with pCiS, pCiM or pCiL, in the presence or absence of pCMVEDEM1. Controls (mock-transfected) cells were also included. Cell lysates were split in two and equal amounts of proteins were subjected to SDS-PAGE under reducing conditions, followed by Western blotting with anti-S (A) or anti-EDEM1 (B) Abs. Calnexin (Cnx) expression was used a total protein, gel loading control.

**Figure 3:**
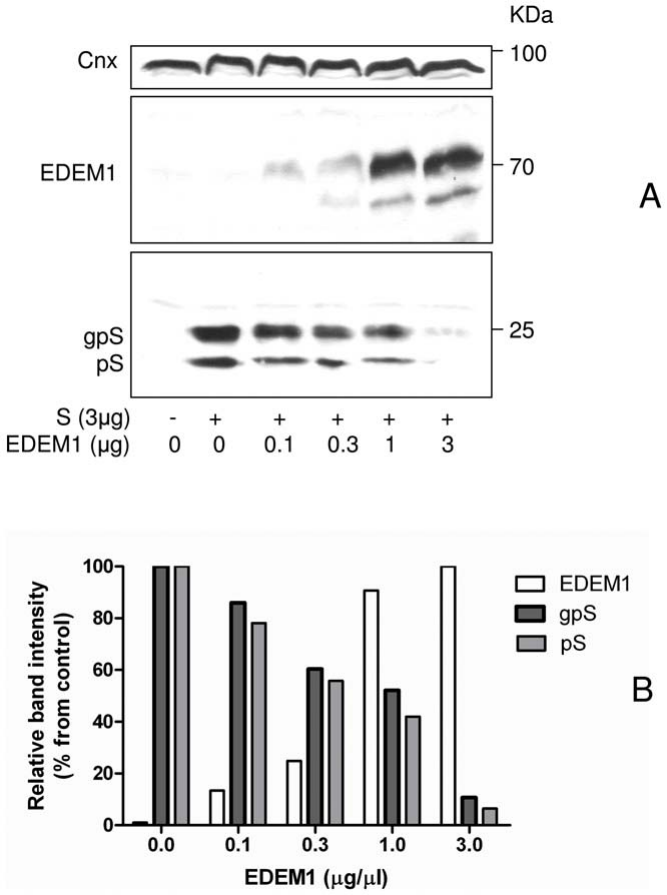
HBV envelope proteins are degraded in the presence of EDEM1, in a dose dependent manner. (A) HEK293T cells were transfected with pCiS in the presence or absence of pCMVEDEM1. Cell lysates were split in two and equal amounts of proteins were subjected to SDS-PAGE under reducing conditions, followed by Western blotting with anti-EDEM (upper panel) or anti-S (lower panel) Abs. Controls, mock-transfected cells were also included. Calnexin (Cnx) expression was used a total protein, gel loading control. (B) The relative band intensities were quantified and compared to control (considered 100%), using the “Quantity One” software from BioRad.

It is generally accepted that EDEM1 function is to relieve the ER stress by removing the burden of misfolded proteins from this compartment. However, wild-type proteins may also be ERAD substrates, as very recently shown for the Human Hepatitis C virus (HCV) E2 envelope protein [16]. To determine whether EDEM1 up-regulation has any consequences on the stability of the HBV envelope proteins, HEK293T cells were co-transfected with plasmids coding for either EDEM1 or the wild-type S, M and L envelope proteins. The steady state level of the viral proteins (Fig. 2A), as well as the expression of EDEM1 in the same cells lysates (Fig. 2B), were assessed by Western blotting using specific Abs. Interestingly, the amount of either envelope protein dramatically decreased in cells co-expressing EDEM1, regardless of their glycosylation status, while the level of the ER chaperone, calnexin, remained unperturbed. This degradation could reflect a function of EDEM1 in the turn-over of the viral proteins or be a consequence of a high, non-physiological level of EDEM1 expression in these cells (Fig. 2B). To investigate either possibility, the stability of the S proteins was analysed in the presence of various amounts of co-expressed EDEM1 (Fig. 3A), followed by quantification using the “Quantity One” software (Fig. 3B). As shown in Fig. 3B, the level of the envelope protein decreased by up to 85% in the presence of EDEM1, in a dose-dependent manner. As little as 0.3 µg of expression plasmid yielding hardly detectable amounts of EDEM1 on Western blots (Fig. 3A) was sufficient to lower the quantity of S proteins by 40%. This dependency strongly suggests a role of EDEM1 in stability of the HBV envelope proteins.

The inverse relationship between EDEM1 expression and HBV may appear to contrast the results in Fig. 1E showing increased expression of both EDEM1 and HBV antigens during transient viral replication in Huh7 cells. In this experimental setting, the amount of viral proteins detected at any time point is the consequence of both, accumulation as a result of genome replication and degradation, as a result of EDEM1 activity. A higher rate of viral replication than of protein degradation may thus explain the apparent discrepancy.

### Knockdown of endogenous EDEM1 increases stability of the HBV envelope proteins

**Figure 4:**
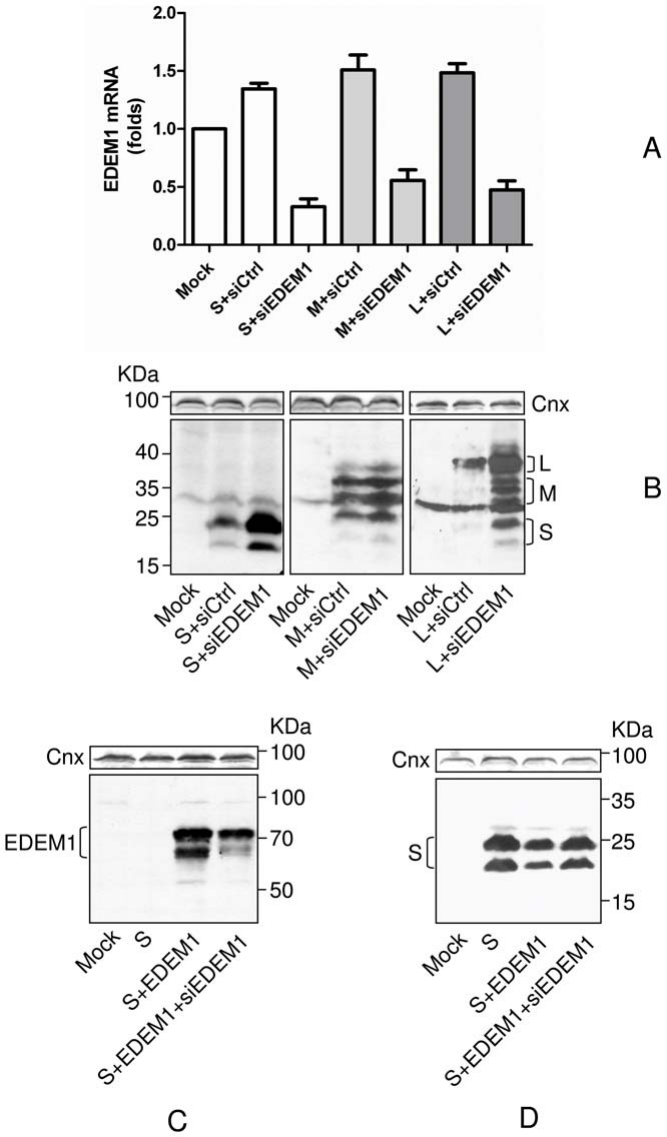
EDEM1 silencing results in inhibition of the HBV envelope protein degradation. HEK293T were transfected with pCiS in the presence or absence of either EDEM1 siRNA (siEDEM1) or scrambled siRNA (siCtrl). Transfected cells were split in equal amounts and analysed for the efficiency of EDEM1 silencing by RT-real-time PCR (A) or the biosynthesis of the S, M and L proteins, by Western blotting using the anti-S Abs. When silencing of the over-expressed EDEM1 was investigated, the pCMVEDEM1 was added in the transfection mixture containing either pCiS or pCiS and EDEM1siRNA, as indicated (D, C). The expression level of EDEM1 (C) and S (D) was determined by Western blotting using the corresponding Abs. Calnexin (Cnx) level was used as total protein-gel loading control.

To further explore the role of EDEM1 in the life cycle of the HBV envelope proteins, expression plasmids coding for S, M and L proteins were co-expressed with EDEM1 siRNA in HEK293T cells. The efficiency of EDEM1 knock down was measured by quantitative RT-PCR in transfected cells, compared to controls. As shown in Fig. 4A, EDEM1 mRNA was significantly down-regulated in cells expressing either S, M or L proteins (by 3 fold), while no cytotoxic effects were observed (data not shown). Analysis of the steady-state levels of the envelope proteins, by Western blotting using the common anti-S Abs, revealed a considerable increase for S and L and more moderate for M, in EDEM1 siRNA-transfected cells (Fig. 4B). It is important to note that in addition to the M protein, the M plasmid also allows for expression of S, due to the presence of the internal ATG start codon. Similarly, S and M are also translated from the L plasmid and are better evidenced in EDEM1 silenced cells (Fig. 4B).

To validate these results, the effect of the over-expressed EDEM1 silencing was also investigated using the S protein as a model substrate. EDEM1 down-regulation and the stability of the S protein were determined by Western blotting, using the corresponding Abs. As shown in Fig. 4C, expression of exogenous EDEM1 was efficiently inhibited, which was accompanied by a clear increase of the envelope protein stability (Fig. 4D).

### EDEM1 interacts with wild-type HBV envelope proteins in HepG2.2.2.15 cells

**Figure 5:**
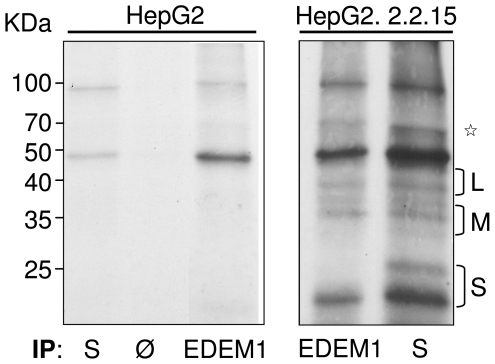
Endogenous EDEM1 interacts with the HBV envelope proteins in HEpG2.2.2.15 cells. HepG2 (control) and HepG2.2.2.15 cells were pulse-labelled for 30 min as described. Cell lysates were split in equal volumes and immunoprecipitated with either anti-EDEM1 or anti-S Abs. Additional control using incubation with Protein A-Sepharose only (Ø) was also included. The protein complexes were resolved by SDS-PAGE followed by autoradiography. The band with the apparent molecular size expected for EDEM1 is marked with an asterisk. The representative autoradiograph of three independent experiments is shown.

EDEMs function in UPR relays on the ability to directly bind ERAD substrates and target them to degradation, thus a potential interaction between EDEM1 and the HBV envelope proteins was further investigated. Conventional co-immunoprecipitation assays using over-expressed proteins, followed by identification of the binding partner by Western blotting did not provide any conclusive results, most probably because of the massive degradation of the viral proteins in the presence of EDEM1 (data not shown). Therefore, an approach whereby the endogenously expressed proteins were radioactively labelled for a short period of time and immunoprecipitation with specific Abs was employed instead. Proteins from HepG2.2.2.15 cells were extracted in mild detergent (CHAPS) and equal amounts were immunoprecipitated with either anti-EDEM1 or anti-S Abs. HepG2 cells and incubation with protein A-Sepharose beads only, were also used as controls. As shown in Fig. 5, no HBV proteins were immunoprecipitated by the anti-S Abs in HepG2 cells, while a very faint band with the apparent molecular size of that expected for EDEM1 (marked with an asterisk) was pulled down in HepG2 cells by the corresponding Abs, but no in the beads only sample (Ø). In contrast, the envelope proteins were co-immunoprecipitated with EDEM1 by both, the anti-S and anti-EDEM1 Abs in HepG2.2.2.15 (Fig. 5, right panel). The ability of EDEM1 to pull down the S, M and L proteins may be the consequence of a direct interaction with each of the three polypeptides, through the common S-domain, or with one of them as part of a homo- or hetero-dimmer. Given the incomplete maturation of the S protein during the short period of pulse-labelling, as measured by the ratio between the glycosylated (g) and nonglycosylated (p) chains, 1∶2 compared to 2∶1 in steady state, the first scenario is more likely to occur. It appears that gpS is not co-precipitated by EDEM1 (Fig. 5, right panel); whether this reflects a lack of interaction between the two proteins, or is simply a consequence of the lower abundance of this glycoform within the cells, is a matter of future investigation.

### HBV envelope proteins are degraded by autophagy

Generally, misfolded proteins are disposed of by the ubiquitin/proteasome ERAD. Interestingly, a mutant variant of the HBV M protein is degraded by an ubiquitin-independent, proteasome-dependent pathway [17]. To investigate the mechanism of the wild-type proteins degradation, the HEK-293T cells expressing the S protein, in the presence or the absence of EDEM1, were treated with a series of proteasome, lysosome and autophagy inhibitors. Soluble tyrosinase (TyrST), a well-studied proteasome-dependent ERAD substrate [18] was included as control for the efficiency of the proteasome inhibition.

**Figure 6:**
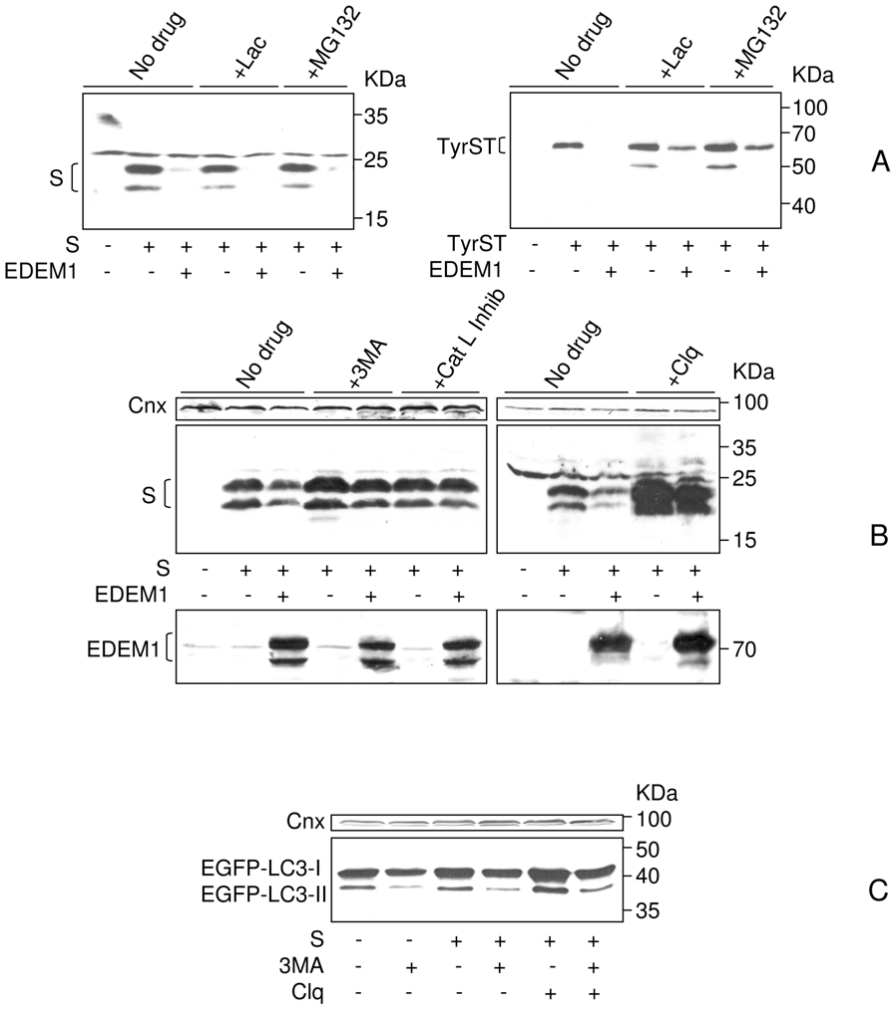
HBV envelope proteins are disposed by autophagy/lysosomal degradation. HEK293T were transfected with pCiS, pTriexTyrST expressing the ERAD substrate soluble tyrosinase (TyrST), or pEGFPC1-LC3 expressing the autophagy marker LC3, in the presence or absence of pCMVEDEM1. The cells were treated with proteasomal (A) or autophagy/lysosome (B, C) inhibitors. Untreated (no drug) cells were also used as control. S, TyrST and EDEM1 synthesis was monitored by Western blotting using the corresponding Abs (A, B). Conversion of EGFP-LC3-I to EGFP-LC3-II was determined by Western blotting using anti-LC3 Abs, in the presence or absence of autophagy inhibitors (C). Calnexin (Cnx) expression was used as total protein-gel loading control.

Interestingly, neither lactacyistin, nor MG132, two classical proteasome inhibitors had any effect in preventing degradation of the S protein (Fig. 6 A, left panel) while efficiently acting on TyrST (Fig. 6A, right panel). In contrast, 3-methyladenine (3MA), an inhibitor of phosphatidylinositol 3-kinase class III (PI3KC3), which is critical for autophagy initiation [19], as well as the cathepsin L inhibitor III, significantly inhibited the S protein degradation, in cells with either endogenous or recombinant EDEM1 expression (Fig. 6B). A similar result was obtained following treatment with the lysosomotropic weak base, chloroquine (Clq).

During autophagy, the Light Chain 3 (LC3)-I protein is lipidated and converted to LC3-II, which is recruited by autophagosome and further degraded within this compartment [20]. This conversion was investigated in the presence and absence of 3MA and Clq, as a control for autophagy. As expected, formation of the faster migrating form LC3-II was inhibited by 3MA, while Clq treatment stabilized this form, when added alone (Fig. 6C). The distribution of LC3 in control and drug-treated cells was also investigated by fluorescence microscopy. As shown in Figure S1, the 3MA treatment was efficient in dispersing the EGFP-LC3 marker from a punctuate pattern characteristic to autophagosome-like vesicles, throughout the entire cytoplasm.

These results strongly indicate that autophagy/lysosomal, rather than proteasomal degradation is involved in disposal of the wild-type HBV envelope proteins.

### EDEM 1 regulates production of HBV virions and subviral particles

**Figure 7:**
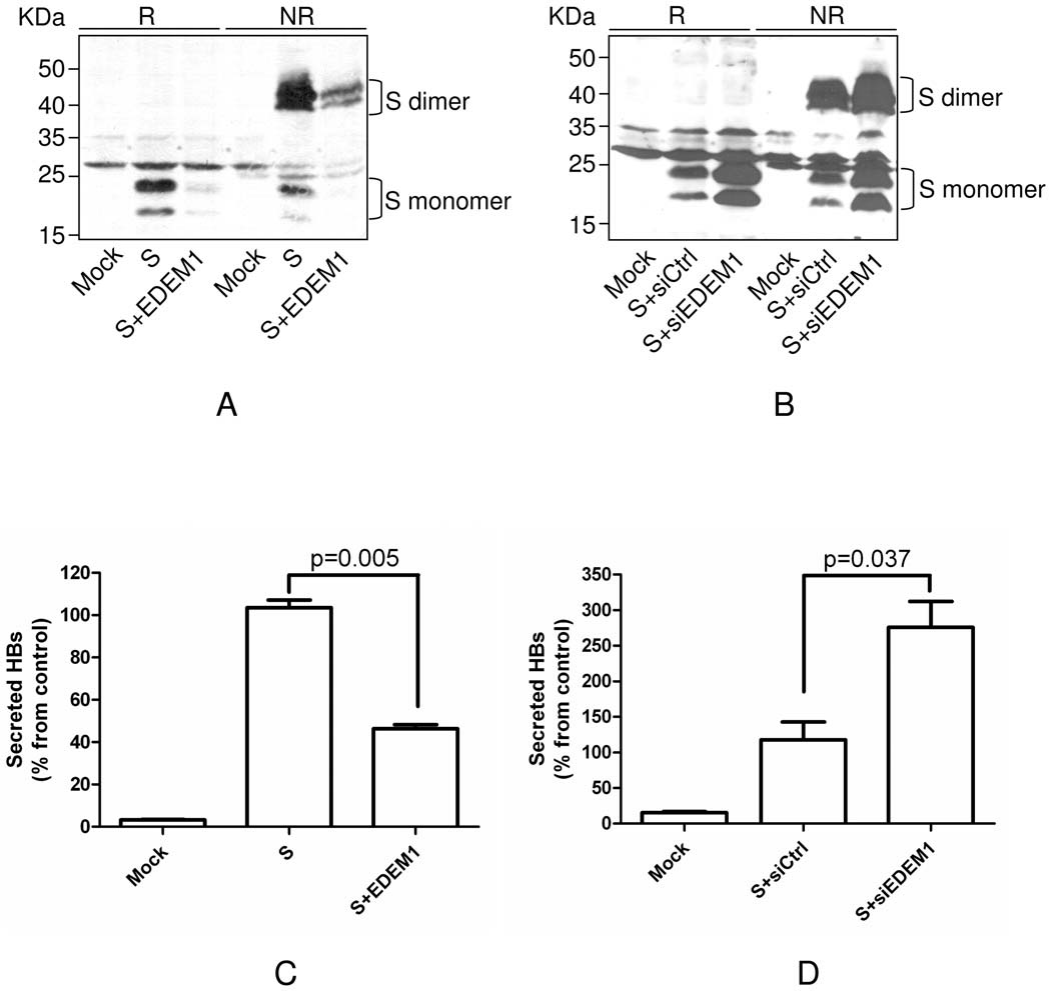
Folding-competent HBV envelope polypeptides are degraded by EDEM1. HEK293T were transfected with pCiS, in the presence or absence of pCMVEDEM1 (A) or EDEM1siRNA (B). Controls, including mock- or scrambled siRNA-transfected (siCtrl) cells were also employed. Both, cell lysates and supernatants were collected at 48 h pt and analysed for the expression S monomers and dimers, by Western blotting under reducing (R) and non-reducing (NR) conditions (A, B) and SVPs secretion, by ELISA (C, D). Note that only the proteins extracted in the soluble phase were loaded on gels (A, B). The cut-off values in ELISA varied between 0.082–0.094. Statistical analysis showing “p” values was performed using the Student's unpaired t-test (C, D).

EDEM1 is clearly involved in the turnover of the wild-type HBV envelope proteins; therefore, it was of interest to determine whether the fraction of proteins that accumulates in EDEM1 knock down cells, or is degraded when EDEM1 is over expressed represents terminally misfolded or folding-competent polypeptides. To investigate these possibilities, the oligomerization status of the S protein was analysed by SDS-PAGE under non-reducing (NR) conditions in cells with either increased or silenced expression of EDEM1. As shown in Fig. 7A, EDEM1 over expression had the same consequence on the amount of both, S monomers and dimmers. Similarly, silencing of endogenous EDEM1 resulted in accumulation of S monomers as well as dimmers, with the same rate (Fig. 7B). It is important to note that the protocol employed in these experiments for cell lysis and protein extraction is not designed to solubilize aggregated complexes. Thus, diffuse bands of higher molecular size are often observed in the upper part of the gels run under NR conditions, most probably as a consequence of protein aggregation (data not shown).

The S protein is the major constituent of SVPs, which are able to assemble and secrete through the normal secretory pathway, even in the absence of M and L co-expression [21]. Thus, secretion of S-containing SVPs was quantified in the presence or absence of EDEM1 expression, as an additional marker of correct protein folding. The amount of S-SVPs considerably decreased (by 50%) when S was co-expressed with EDEM1 in HEK-273T cells (Fig. 7C). More importantly, rescuing the S protein from degradation, by silencing endogenous EDEM1, resulted in a significant increase of S-SVPs in cell supernatant (by 2 fold) (Fig. 6D).

**Figure 8:**
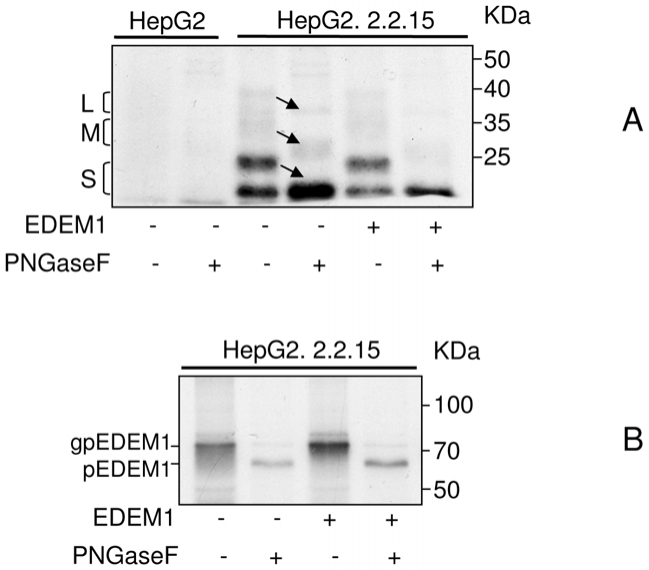
HBV SVPs secretion from HepG2.2.2.15 cells is inhibited in the presence of EDEM1. HepG2 (control) and HepG2.2.2.15 were grown in 6-well plates and transfected or not with pCMVEDEM1. Cells were further pulse-labelled for 4 h, as described. (A) The cell medium was collected and immunoprecipitated with anti-S Abs and the bound proteins were split in two and subjected or not to PNGase F digestion. The arrows mark the shift of the L, M and S glycoforms towards the corresponding deglycosylated polypeptides, in the presence of PNGase F. (B) The HepG2.2.2.15 cells were lysed and immunoprecipitated with anti-EDEM1 Abs. The bound proteins were split in two and subjected or not to PNGase F digestion. Glycosylated (gp) and non-glycosylated (p) EDEM1 are shown.

The effect of EDEM1 over-expression was also investigated on SVPs, in the context of full viral replication, assembly and secretion. EDEM1-transfected HepG2.2.15 cells were subjected to a long radioactive pulse-labelling (4 hr) and secreted SVPs were immunoprecipitated form cell supernatants and digested with PNGase F to better reveal the identity of the constituent polypeptides (Fig. 8A). The intracellular level of endogenous as well as over-expressed EDEM1 was also determined by immunoprecipitation with the corresponding Abs followed by PNGase F treatment, showing an inverse correlation between EDEM1 levels and the amount of secreted SVPs. (Fig. 8B).

Altogether, these results strongly suggest that EDEM1 induces a premature extraction of the wild-type viral envelope proteins from the folding cycle, promoting degradation of otherwise folding-competent polypeptide chains.

**Figure 9:**
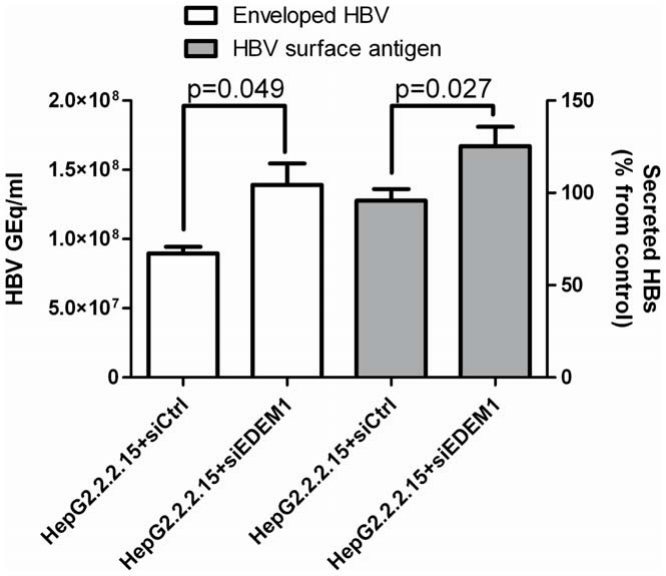
EDEM1 silencing results in increased secretion of enveloped HBV. HepG2.2.2.15 cells were transfected twice with either EDEM1 siRNA (siEDEM1) or scrambled (siCtrl) siRNA. At 3 days pt, cell medium was collected and either immunoprecipitated with a mixture of anti-S/preS1Abs (white bars) or analysed for secretion of SVPs by ELISA (grey bars). The enveloped viral particles were further quantified following DNA purification and real-time PCR. The cut-off values in ELISA varied between 0.085–0.096. Statistical analysis showing “p” values was performed using the Student's unpaired t-test.

Under physiological conditions SVPs greatly outnumber the viral particles; it was thus possible that, despite significantly contributing to the degradation of the envelope proteins, EDEM1 may have no effect on HBV envelopment. To investigate this hypothesis, endogenous EDEM1 was silenced in HepG2.2.2.15 cells and the amount of fully enveloped virions was immunoprecipitated from cell supernatant using a mixture of anti-S and anti-PreS1 Abs, followed by quantification by real time PCR. Secreted SVPs were also measured by ELISA, in the same supernatants. As shown in Fig. 9, the number of enveloped virions secreted from EDEM1-silenced cells was by almost 2 fold greater than in cells treated with scrambled siRNA. Similarly, the amount of SVPs increased by about 35%, as compared to controls, suggesting a rather direct effect of EDEM1 silencing on production of both viral proteins and virions. However, as the stability of other intracellular substrates, with potential role in HBV assembly and secretion, may also depend on the EDEM1 level, an indirect effect of the siRNA treatment on HBV cannot be totally excluded.

Together, the results imply that EDEM1 up-regulation during HBV infection has an important function in controlling the level of virions secreted from cells.

## Discussion

Whilst many studies have implied a function of ERAD in various pathologies, known as “conformational diseases”, including inflammation, diabetes, and neurodegenerative disorders [22], [23], the relationship between ERAD and viral infection and pathogenesis was very little investigated. Several viral infection were shown to induce UPR [24], [25] and very recently, an important role of the ERAD pathway in modulating production of infectious HCV, but not of other viruses from the same family, was clearly demonstrated, suggesting a high specificity, with physiological consequences for certain viruses [16].

Here we show that HBV life cycle and the ERAD component EDEM1 are strongly interconnected. EDEM1 expression was significantly up-regulated in cells replicating HBV either stably or transiently, as a result of viral protein expression and independent of replication and nucleocapsid accumulation. Interestingly, expression of each of the envelope proteins was on its own sufficient to induce synthesis of EDEM1 mRNA in HEK cells, however, at a more moderate level compared to HepG2.2.2.15 cells. Most likely this is due to the presence of the HBV X protein in HepG2.2.2.15 cells, a regulatory protein recently linked to activation of the IRE1 branch of the UPR in these cells [12].

Manipulating the EDEM1 level by either over-expression or silencing experiments clearly demonstrated a role in the stability of the wild-type HBV envelope proteins and thus, in controlling the amount of native polypeptides available for SVPs assembly and virion envelopment. Analysis of the steady-state level of the envelope proteins, under non-reducing conditions, showed that EDEM1 acts during, or immediately post-translation, likely before polypeptide assembly into oligomers. It is important to note that degradation of S, M and L proteins occurred regardless of their glycosylation status, implying no role for the N-linked glycans in this process. Also, endogenous EDEM1 was able to co-precipitate the glycosylated as well as non-glycosylated viral polypeptides, suggesting a direct interaction, possibly through the common S-domain. These results are in agreement with the more recent model describing the molecular details of substrate recognition by EDEM1, whereby specific binding of the ERAD targets occurs in a glycan-independent manner [26]. However, the model also implies that EDEM1 is able to discriminate between aberrantly folded and native proteins during polypeptide maturation and quality control. In contrast, here we provide clear evidence that EDEM1 acts on and promote degradation of the wild-type envelope proteins encoded by HBV, with important implications for the viral life cycle. This effect was not due to the over-expression of the viral proteins in a heterologous system, as similar degradation rates were observed in cells allowing for both, high and moderate level of viral protein synthesis (HEK293T and HepG2.2.2.15, respectively). The envelope proteins rescued from degradation in endogenous EDEM1-knock down cells were able to assemble in functional, secretion-competent subviral particles, implying that they do not originate from a pool of terminally misfolded polypeptides. Moreover, these proteins are actively recruited for nucleocapsid envelopment, which suggest that virion and SVPs assembly may be competing processes, despite the envelope proteins being synthesised in vast excess.

Interestingly, it was recently found that up-regulation of EDEM1, a condition occurring during UPR, bypasses the requirement for mannose trimming of glycoprotein ERAD substrates. Consequently, the folding quality control “check-point” regulated by EDEM1 is canceled and nascent glycoproteins are delivered directly to late ERAD stages, regardless of their conformation status [27]. It is tempting to speculate that the significant activation of EDEM1 expression in HBV-replicating cells may cause a similar effect, promoting premature extraction of folding competent viral glycoprotein intermediates from the “calnexin cycle” and their subsequent degradation. As a direct consequence, SVP and more importantly, virion production is lowered, thus contributing to HBV persistence in chronic infections.

Strong evidence in support of this hypothesis come form the recent HCV studies showing that EDEM1 is involved in the post-translational control of wild-type envelope glycoproteins, by which viral production is down-regulated. EDEM1 silencing, as well as cell treatment with proteasome inhibitors, resulted in increased stability of the E2 protein and enhanced secretion of infectious virions, while the viral replication rate remained unchanged [16].

Unlike HCV, the wild-type envelope proteins encoded by HBV appear to be degraded by autophagy/lysosmes. Autophagy usually plays a role in the degradation of long-lived proteins and damaged cellular organelles under critical conditions, such as nutrient stress. However, constitutive autophagy has been recently involved in maintaining the homeostasis of non-dividing cells, including hepatocytes and neural cells [28], [29]. In yeast, autophagy can function along with the ubiquitin/proteasome system, as mechanistically distinct degradation pathways used to remove proteins from the ER, as shown for two variants of an α-1 proteinase inhibitor [30]. Two ERAD models were also recently proposed for disposal of dysferlin, a type-II transmembrane protein involved in muscular dystrophy type 2B and Miyoshi myopathy [31]. Usually, wild-type and mutant dysferlin are degraded by the ubiquitin/proteasome system - ERAD (I); however, when this degradation is not sufficient, the proteins aggregate on the ER membrane and stimulate autophagy formation. Thus, the autophagy/lysosome degradation - ERAD (II) may function as an alternative pathway when the retrotranslocon/ERAD (I) system is impaired by the accumulation of protein aggregates within the ER.

In the case of HBV, correctly folded envelope polypeptides become highly cross-linked by disulfide-bonds within the ER, before assembly into virions and SVPs. Therefore, it is possible that the unstable polypeptides, prematurely extracted from the quality control cycle when EDEM1 is up-regulated, are forced into aberrant intra- and inter-molecular disulfide bond pairing and further aggregation.

Although the exact mechanism of the HBV wild-type envelope protein degradation is not clear at the moment, it is important to note that HBV infection is able to induce autophagic response, which in turn, enhances viral DNA replication and supports nucleocapsid envelopment [13], [32]. In this context, degradation of the envelope proteins using the same pathway may appear as a compensatory mechanism to regulate the level of virions, reduce cellular stress and establish persistent infections.

## Materials and Methods

### Cell lines, inhibitors and enzymes

Huh7, HepG2 and HEK 239T cells (European Collection of Animal Cell Culture, Porton Down, UK) were grown in RPMI 1640 medium (Euroclone) containing 10% fetal bovine serum (FBS), 50 units/ml penicillin, 50 µg/ml streptomycin and 2 mM Glutamax (Invitrogen). HepG2 2.2.15 cells (kind gift from Dr. Durantel D., INSERM U871, Lyon, France), stably transfected with two copies of the HBV genome, were grown as above, except that the RPMI medium was supplemented with 200 µg/ml of G418 (Gibco). Lactacystine (Lac) was from Toronto Chemicals, MG132 was from Santa Cruz, Tunicamicin (Tu) was from MP Biomedicals, 3-methyladenine (3MA) and chloroquine (Clq) were from Sigma-Aldrich and the cathepsin L inhibitor III (Cat L Inhib) was from Calbiochem. The peptide N-glycanase F (PNGase F) was from New England Biolabs (UK).

### Plasmids and siRNA

Plasmids pCiS, pCiM or pCiL coding for S, M and L proteins, containing the CMV enhancer/promoter and the late SV40 polyadenylation sequences were a kind gift from Dr. Durantel D., INSERM U871, Lyon, France. pTriExHBV 1.1 containing 1.1 units of the whole HBV genome, supporting viral replication, assembly and secretion of fully infectious virions was described previously [15]. The plasmid pCMVEDEM1 expressing the mouse EDEM1 under the control of the CMV promoter was a kind gift from Dr. Nagata K., Kyoto Sangyo University, Kyoto, Japan. The plasmid pTriExTyrST expressing the human tyrosinase without the transmembrane domain was previously described [18]. The plasmid pEGFPC1-LC3 expressing the the autophagy marker LC3, N-terminally fused with the Enhanced Green Fluorescence protein (EGFP), under the control of the CMV promoter, was a kind gift from Dr. Tamotsu Yoshimori, National Institute of Genetics, Mishima, Japan. The siRNA designated to silence EDEM1 expression and control siRNA (siCtrl) were purchased from Santa Cruz and contain a pool of 3 target-specific 20–25 oligonucleotides (sc-43745) or a scrambled sequence (sc-37007), respectively.

### Cell tranfection and gene silencing

Monolayers of HEK293T or HepG2.2.2.15 cells (80% confluence) were transfected with 3 µg of plasmid and/or 50 nM siRNA using Lipofectamine 2000 (Invitrogen). At 24 h post-transfection (pt) the HEK cells were treated with Lac (20 µM), MG132 (25 µM), 3MA (5 mM), Cat L Inhib (25 µg/ml), Clq (100 µM) or were left untreated. After another 24 h, the cells and supernatants were harvested. The HepG2.2.2.15 cells were transfected twice, at 24 h interval. The cells and supernatants were harvested 3 days pt.

### SDS-PAGE and Western blotting

Transfected or mock transfected (control) cells were lysed in a buffer containing 10 mM Tris-HCl (pH 7.5), 150 mM NaCl, 2 mM EDTA, 0.5% TritonX-100 and a mixture of protease inhibitors (Sigma-Aldrich) for 1 h, on ice. Lysates were clarified by centrifugation for 10 min at 10,000× g and the protein content was determined in supernatant using the BCA method (Pierce). Equal amounts of total proteins in the supernatant were either boiled under non-reducing conditions (NR) or boiled in the presence of 5 mM DTT (reducing conditions-R) before SDS-PAGE and Western blotting. Where indicated in the figures, samples were digested with PNGase F, following the protocol supplied by the manufacturer. The proteins were transferred to nitrocellulose membranes using a semi-dry blotter (Millipore). The blots were incubated with mouse anti-preS1 (Santa Cruz, dilution 1/1000), rabbit anti-S (Europa Bioproducts, dilution 1/1000), goat anti-calnexin (Santa Cruz, dilution 1/1000), mouse anti-β actin (Abcam, dilution 1∶500), mouse anti-LC3 (NanoTools, dilution 1∶1000), or rabbit anti-EDEM1 (home-made, dilution 1/1000) antibodies (Abs) followed by anti-mouse (Santa Cruz, dilution, 1/10,000), anti-rabbit (Santa Cruz, dilution 1/10,000) or anti-goat (dilution 1/10,000) Abs conjugated to horseradish peroxidase. The proteins were detected using an enhanced chemiluminiscence (ECL) detection system (Amersham, UK) according to the manufacturer's instructions.

### Quantification of HBV secretion by immunoprecipitation and real-time PCR

Transfected HepG2.2.2.15 cells were grown for 3 days and 500 µl of medium was used for immunoprecipitation with a mixture of mouse anti-preS1 (dilution 1∶500) and rabbit anti-S (dilution 1∶500) Abs. The immune complexes were isolated by incubation of samples with protein A- Sepharose beads (Sigma), over-night, at 4°C. In control samples, cell lysates were incubated with beads only. The slurry was washed five times with PBS and the bound virions were eluted by boiling the samples for 10 min, in 50 mM Tris-HCl buffer (pH 8) supplemented with 1 mM EDTA and 1% NP40, with strong shaking. Encapsidated viral DNA was purified form supernatants by phenol-chloroform extraction, as described elsewhere [33]. The DNA was quantified using the Corbett Rotor Gene 6000 real-time PCR system and the Maxima SYBR Green qPCR Master Mix (Fermentas). Primers were designed to amplify a 279 bp (see Table S1). The number of viral genome equivalents was determined using a calibration curve containing known amounts of HBV DNA.

### Quantification of HBV replication by Southern blotting

HepG2 or HepG2.2.2.15 cells were used to purify the encapsidated viral DNA by phenol-chloroform extraction, as for the real-time PCR. The resulting DNA pellet was resuspended in nuclease-free water, analyzed in a 1.2% agarose gel and transferred to a Hybond-N+ membrane (GE Healthcare), using a vacuum transfer blotter (BioRad). The blot was further hybridized with a fluorescein-labelled probe obtained by random priming using the HBV DNA genome as template. The HBV-specific DNA bands were detected using anti-fluorescein alkaline phosphatase (AP)-conjugated monoclonal antibodies (MAbs) and the Gene Images CDP-Star detection kit (GE Healthcare).

### Quantification of HBV SVPs secretion by ELISA

Supernatants from Huh7, HEK293T or HepG2.2.2.15 transfected cells were analysed for the amount of secreted HBsAg, using the Monolisa HBsAg Ultra Kit (Bio-Rad). The results were obtained as ratios of signal to cut-off value and were converted to percentages of HBsAg secretion.

### Quantification of gene expression by reverse transcription (RT)-real-time PCR

Total RNA from HepG2, HepG2.2.2.15 or Huh7 transfected cells was isolated using RNeasy mini kit (Qiagen). The RNA was quantified using a Corbett Rotor Gene 6000 real-time PCR system and the SensiMix One-Step Kit (Quantance). Primers were designed to amplify either HBV- or EDEM1-3- specific fragments (Table S1). For viral quantification, a calibration curve containing known amounts of HBV was used. The values obtained were standardized against an internal β-actin control. Where indicated in the figure legends cells were also treated with either 10 µM 3TC or 5 µg/ml tunicamycin.

### Pulse-labeling and immunoprecipitation

Subconfluent HepG2 and HepG2.2.2.15 cells grown in six-well plates were were labelled as described before (as described [34]. Briefly, the monolayers were washed once with PBS and incubated in methionine- and cysteine-free RPMI 1640 medium (ICN Flow). After 1 h, the cells were pulse-labeled with 100 µCi of ^35^Smethionine-^35^Scysteine (Tran ^35^S-label, 1,100 Ci/mmol; ICN Flow) per ml at 37°C for the times indicated. The cells were lysed under mild conditions using a CHAPS-HSE buffer (2% CHAPS in 50 mM HEPES, pH 7.5, 200 mM NaCl, 2 mM EDTA). Labelled cell lysates were clarified by centrifugation as described before. The supernatants were incubated with either anti-EDEM1 or anti-S Abs (diluted 1∶50 and 1∶100, respectively), overnight at 4°C. Protein A-Sepharose (30 µl) was then added, and the incubation continued for 1 h at 4°C. The slurry was washed 5 times with 0.5% CHAPS-HSE buffer and the bound complexes were eluted by boiling the samples for 10 min in SDS-PAGE sample buffer. SVPs were immunoprecipitated in the cell medium using the anti-S Abs and the same protocol as above. When PNGase F digestion was employed, the bound proteins were eluted by boiling the samples for 10 min in 1% SDS, followed by addition of the enzyme reaction buffer and over-night incubation at 37°C. Labelled proteins were visualised by SDS-PAGE and analyzed by autoradiography.

## Supporting Information

Figure S1 **3MA treatment of EGFP-LC3-transfected HEK293T cells results in LC3 dispersion from punctuate autophagosome-like vesicles throughout the cytoplasm.** HEK293T cells were transfected with pEGFPC1-LC3. At 24 h post-transfection cells were treated with 5 mM 3MA for 12 h, then either nutrient starved in the presence of Earl's buffer (140 mM NaCl, 5 mM KCl, 1.8 mM CaCl_2_, 0.9 mM MgCl_2_, 25 mM HEPES, pH 7.4) or incubated with 100 mM chloroquine (Clq), for 4 h. EGFP-LC3 expression and DAPI-stained nuclei were evidenced by fluorescence microscopy with a Nikon E600 fluorescence microscope (60× magnification).
(TIF)

Table S1 **Sequences of primers used for quantification by RT-real-time PCR or real-time PCR.**
(DOC)
