# Supplementary figures and images for: Activation of ERAD Pathway by Human Hepatitis B Virus Modulates Viral and Subviral Particle Production
Source: PLoS One. 2012 Mar 26;7(3):e34169. doi: 10.1371/journal.pone.0034169 (PMC3312915; doi:10.1371/journal.pone.0034169)

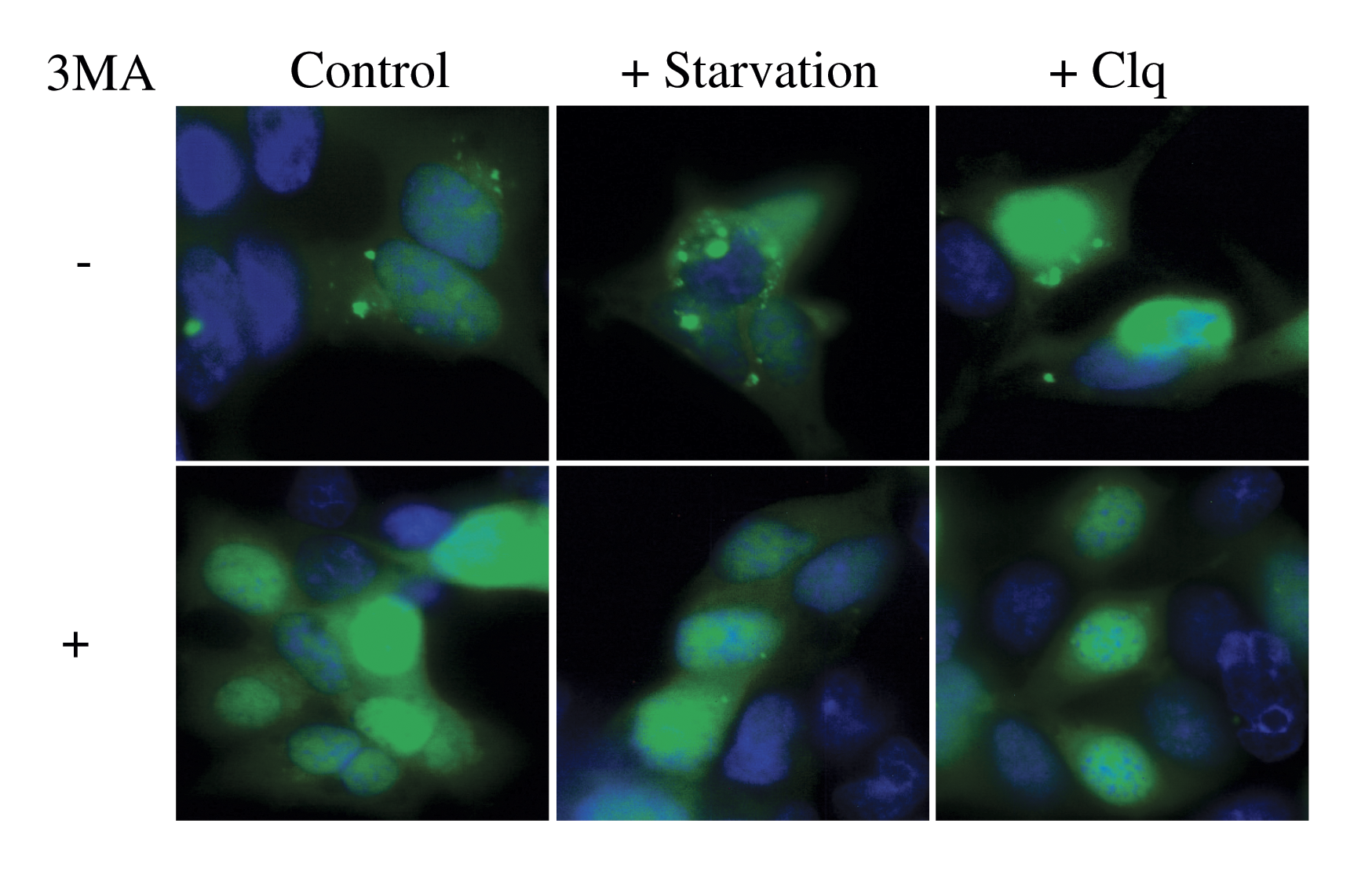

Supplement: Figure S1 — 3MA treatment of EGFP-LC3-transfected HEK293T cells results in LC3 dispersion from punctuate autophagosome-like vesicles throughout the cytoplasm. HEK293T cells were transfected with pEGFPC1-LC3. At 24 h post-transfection cells were treated with 5 mM 3MA for 12 h, then either nutrient starved in the presence of Earl's buffer (140 mM NaCl, 5 mM KCl, 1.8 mM CaCl2, 0.9 mM MgCl2, 25 mM HEPES, pH 7.4) or incubated with 100 mM chloroquine (Clq), for 4 h. EGFP-LC3 expression and DAPI-stained nuclei were evidenced by fluorescence microscopy with a Nikon E600 fluorescence microscope (60× magnification). (TIF) [file pone.0034169.s001.tif]
