# Supplementary material for: Activation of ERAD Pathway by Human Hepatitis B Virus Modulates Viral and Subviral Particle Production
Source: PLoS One. 2012 Mar 26;7(3):e34169. doi: 10.1371/journal.pone.0034169 (PMC3312915; doi:10.1371/journal.pone.0034169)
Supplement: Table S1 — Sequences of primers used for quantification by RT-real-time PCR or real-time PCR. (DOC) [file pone.0034169.s002.doc]

|  | Forward primer (5’-3’) | Reverse primer (5’-3’) |
| --- | --- | --- |
| HBV | TCCAGGATCCTCAACAACCAGCACG | TGGCCCCCAATACCACATCATCC |
| EDEM1 | TCCTTAAAGGGGAAGCGAGCC | AGCGCTCGCCATTGCATGGT |
| EDEM2 | AGTGGTTGAAGTGCTCCAGGA | CAGCCTCTACTTCCACCCCA |
| EDEM3 | GGCTTGGTGGCTTCGGGAAA | ACATTGCTGGACGCTGGTGG |
| Actin | GAAAATCTGGCACCACACCTTC | CTCGGTGAGGATCTTCATGAGG |
